# Under-Representation of Women on Dental Journal Editorial Boards

**DOI:** 10.1371/journal.pone.0116630

**Published:** 2015-01-30

**Authors:** Effie Ioannidou, Amy Rosania

**Affiliations:** 1 Division of Periodontology, Dental Clinical Research Center, University of Connecticut Health Center, Farmington, Connecticut, United States of America; 2 Advanced Education Program in Periodontology, Division of Periodontology, University of Connecticut Health Center, Farmington, Connecticut, United States of America; State University of New York, Oswego, UNITED STATES

## Abstract

**Introduction:**

Each journal’s editorial and advisory board plays a critical role in resolving gender bias in the peer-review and publication process. Thus, this study aimed to quantify women’s participation in editorial and advisory boards of major dental journals. Gender data on editorial and advisory boards were extracted from major dental journals, which were then categorized by journal specialty focus. The gender of the editor-in-chief and associate editor-in-chief was noted to assess the effect of journal leadership on women’s participation in journal boards. For comparison purposes, data were also obtained regarding the percentage of women faculty for each dental specialty.

**Results:**

Overall, in the major 69 dental journals, 14.8% of editorial board members were women. An one-way ANOVA analysis revealed statistically significant gender differences between journal specialty categories (p = 0.003) with some dental specialties’ journals demonstrating a relatively high participation of women as editorial board members. There was a significant positive correlation for various dental specialties between women’s representation in editorial and advisory boards and women in similar dental academic specialties (p = 0.02, r^2^ = 0.55). Furthermore, there was a positive correlation between the presence of women in journal editorial leadership and the percentage of women serving as advisory board members (p = 0.03). Our results confirmed that the under-representation of women on dental journal editorial boards was significantly different between dental science specialties. When there were more women in journal editorial leadership positions, there was a higher participation of women as editorial and advisory board members. Journals should increase the numbers of women on editorial boards in order to secure diversity, improve publication quality and recognize women’s contribution to dental science.

## Introduction

Despite the social and civil gains achieved by women in the 20^th^ century, there is still a gender gap in academia [1–3]. Evidence has shown that women in academia are less likely to achieve tenure, are expected to meet higher standards than male counterparts, and frequently exit tenured positions for adjunct ones [4,5]. Women frequently perceive the academic environment as hostile which leads to high attrition rates and low job satisfaction [1]. Women often face social and personal dilemmas in balancing career and family without any substantial institutional support [6].

Additional struggles for women in academia have been reported in authorship [7,8] and participation on editorial and advisory boards [9–15]. Limited evidence demonstrates gender inequalities in authorship of dental publications [16–18]. Each journal’s editorial and advisory board plays a critical role in resolving gender bias in the peer-review and publication process. The National Academy of Sciences, in their *Beyond Bias and Barriers* report, calls for the “reasonable representation of women on editorial boards” [1]. Our study assessed the level of women participating on editorial and advisory boards of major dental journals. In addition, we quantitatively compared the participation of women on editorial and advisory boards with the percentage of women in the corresponding dental academic specialties.

## Methods

### Journal Selection and Stratification

The first activity of this cross-sectional study was the selection of dental journals based on the list in Thomson Reuters Web of Knowledge Journal Citation Reports (2012) under the category of “Dentistry, Oral Surgery and Medicine” [19]. The search was limited to English language journals. Also, journals specializing in dental hygiene were excluded in order to control for bias that would be derived from the overrepresentation of women in the hygiene field.

Once the journal list was compiled, each individual journal homepage was searched for gender identification of the editor-in-chief as well as the editorial and advisory board members. Editors-in-chief and associate editors-in-chief were considered the editorial leadership of each journal. The gender of the journal leadership and board members was identified based on first-name recognition. When the first-name gave no clues to the gender, an internet search of the individual was used to identify gender. In cases where gender could not be identified, the journal’s editor-in-chief was contacted via email twice for additional clarifications.

The journals were then stratified into the following 10 categories based on the specialty concentration field: endodontics, general dentistry, oral medicine/oral pathology and radiology, oral and maxillofacial surgery, orthodontics, pediatric dentistry, periodontology, prosthodontics, public health, and research.

Data on women faculty per dental academic sub-field were extracted from the American Dental Education Association (ADEA) Faculty Survey[20] and were used as an indicator of women representation in dental academic specialties in the US.

### Statistical Analyses

Statistical analysis was performed with the PASW Statistics, version 18. All variables were tested for normality. Any non-normally distributed variable was logarithmically transformed. Means and standard deviations were calculated and compared with one way analysis of variance (ANOVA) followed by a post hoc Fisher’s least significant differences (LSD) analysis when appropriate. The correlation of women faculty per specialty and women representation in each journal category was tested by Pearson’s correlation test. This analysis was based on the ADEA Faculty Survey and limited to US published journals. We further assessed the association between the journal leadership (editor-in-chief and associate editor-in-chief) and the participation of women on journal advisory boards with correlation analyses. A p-value less than 0.05 indicated statistical significance.

## Results

**Figure 1:**
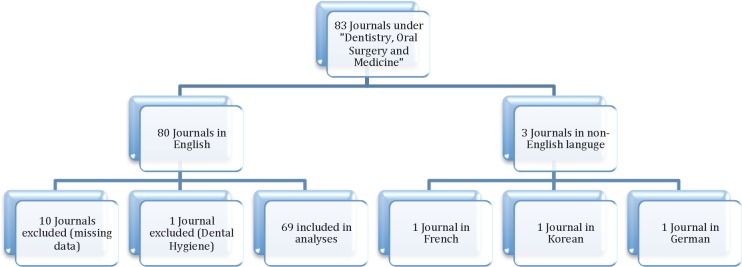
Flow chart of journal selection based on inclusion/exclusion criteria.

A total of 83 journals were included in the Journal Citation Reports under the category of “dentistry, oral surgery and medicine”. When limiting the search to English language journals, 70 journals were included. After completing the electronic search and receiving email responses from journals, 69 journals had adequate data and were included in the analyses (Fig. 1).

Out of the 3060 editorial board members, 452 were women (14.8%) ranging from zero to 57.1%. Overall, women represented 2.5% of the editors-in-chief and 16.0% of the associate editors-in-chief.

**Figure 2:**
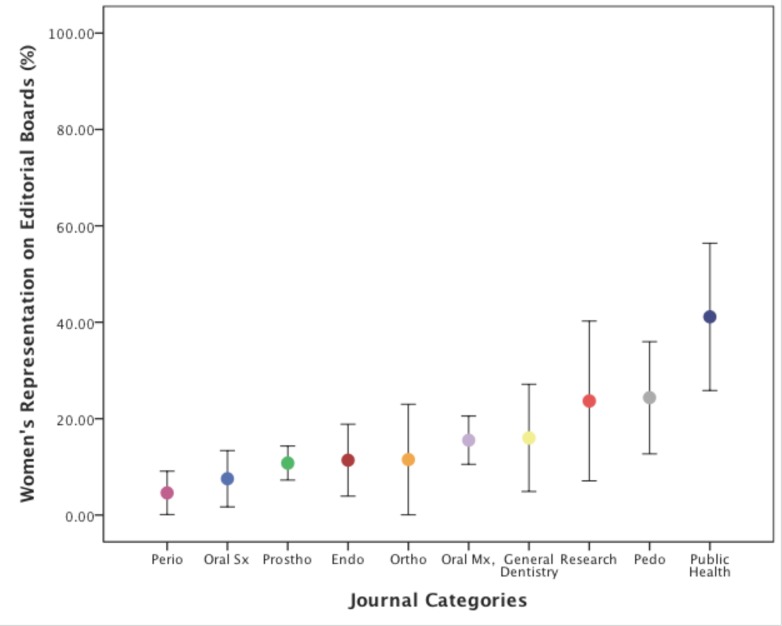
Women’s representation on Dental Journal Editorial Boards. Journal Categories in an ascending order based on statistical mean (mean and standard deviation, as represented by solid circle and bards, respectively). Periodontology journals showed the lowest proportion of women on editorial boards followed by oral and maxillofacial surgery. Public health journals showed the highest representation of women in editorial boards. Perio: periodontology journals, Oral Sx: oral and maxillofacial surgery journals, Prostho: prosthodontic journals, Endo: endodontic journals, Ortho: orthodontic journals, Oral Mx: oral medicine, oral pathology, oral radiology journals, Pedo: pediatric dentistry journals.

Table 1 shows the descriptive analysis per journal category. Skewness was assessed and all variables were deemed normally distributed. Fig. 2 graphically shows women’s representation as expressed by mean and standard deviation in statistical ascending order.

**Table 1:** Descriptive statistics of women’s participation on editorial/advisory boards and editorial leadership per dental specialty.

**Journal Categories**	**Journals #**	**Women Editorial Board Members (%) Mean±SD (Range)**	**Women on Editorial Leadership (%) Mean±SD (Range)**	**Women Faculty (%)**
**Endodontics**	3	11.4±7.5 (7.0–20.0)	19.7±26.6 (0.0–50.0)	13.9
**General Dentistry**	11	16.0±11.1 (0–33.3)	13.0±22.3 (0.0–50.0)	28.9
**Oral Medicine, Oral Pathology, Oral Radiology**	6	15.5±5.0 (7.89–21.31)	17.5±14.3 (2.48–32.50)	36.1
**Oral Maxillofacial Surgery**	9	7.5±5.8 ^§, ⌘, ¥^(0.0–16.3)	0.9±2.8 (0.0–8.3)	7.0
**Orthodontics**	5	11.5±11.5 (0.0–26.7)	10.5±17.4 (0.0–40.0)	17.8
**Pediatric Dentistry**	6	24.3±11.6 (6.7–38.5)	31.8±38.4 (0.0–100.0)	32.5
**Periodontology**	6	4.6±4.5^§, ¶, ¥^(0.0–11.1)	3.3±7.5 (0.0–16.7)	22.0
**Prosthodontics**	7	10.8±3.5 (5.1–16.5)	4.04±6.41 (0.0–14.3)	17.1
**Public Health**	4	41.1±15.3 (18.3–50.0)	14.28±28.57 (0.0–57.1)	41.2
**Research**	12	23.7±16.7 (0.0–56.3)	14.0±17.8 (0.0–50.0)	NA

Journals are alphabetically ranked. Women academic faculty data are shown (Source: ADEA, 2010–11 Comprehensive Faculty Salary Survey). One-way ANOVA showed a significant difference between specialties (p = 0.003). Post hoc analysis revealed statistically significant differences between the journal in the field of periodontology and oral and maxillofacial surgery as compared to §: public health Journals (p = 0.001), ⌘: pediatric dentistry journals (p = 0.004), ¥: research journals (p = 0.001), ¶pediatric dentistry journals (p = 0.002). SD: Standard Deviation.

In the between-group analysis, the one-way ANOVA revealed a statistically significant difference between journal categories (p = 0.003). More specifically, periodontology and oral and maxillofacial surgery were the two specialties with the lowest representation of women on journal editorial boards ranging from 0–16.3%. On the other end of the spectrum, public health journals showed the highest women’s representation on editorial boards (41.1% average).

In a post-hoc statistical analysis, women’s representation on editorial and advisory boards was demonstrated to be significantly less in periodontology journals compared to journals of public health (p = 0.001), pediatric dentistry (p = 0.002) and research (p = 0.001). Moreover, women’s representation on editorial board of the oral and maxillofacial surgery journals was significantly less than journals pertaining to public health (p = 0.001), pediatric dentistry (p = 0.004) and research (p = 0.001).

**Figure 3:**
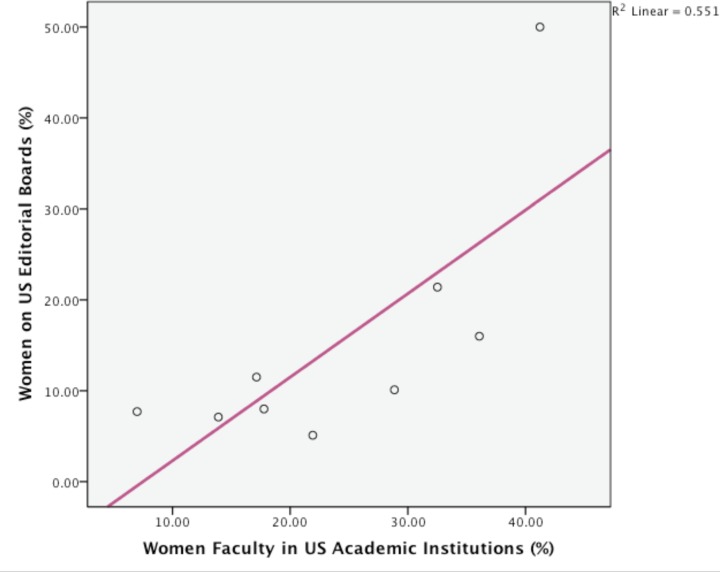
The association of women on editorial/advisory board and in the corresponding academic specialty. The scatterplot and fit line shows a linear trend (r^2^ = 0.55) and confirms a positive and significant correlation between women on editorial and advisory boards and women faculty per academic specialty (p = 0.01).

The data on women faculty representation per specialty, as reported by ADEA, are included on Table 1. The ADEA data confirmed a statistically significant difference in women distribution in academic specialties (p = 0.03). In addition, when the association between women participation on editorial/advisory boards and women faculty in US academic disciplines was tested, a statistically significant correlation was identified (p = 0.02, r^2^ = 0.55) (Fig. 3).

There were only two journals identified that had women as editors-in-chief. For these two journals, the average representation of women on the advisory board was 11.3% whereas journals with men as editor-in-chiefs, the average participation of women on the advisory board was higher at 16.4%. With only two women editors-in-chief, the sample size was too small to allow any additional statistical comparisons.

**Figure 4:**
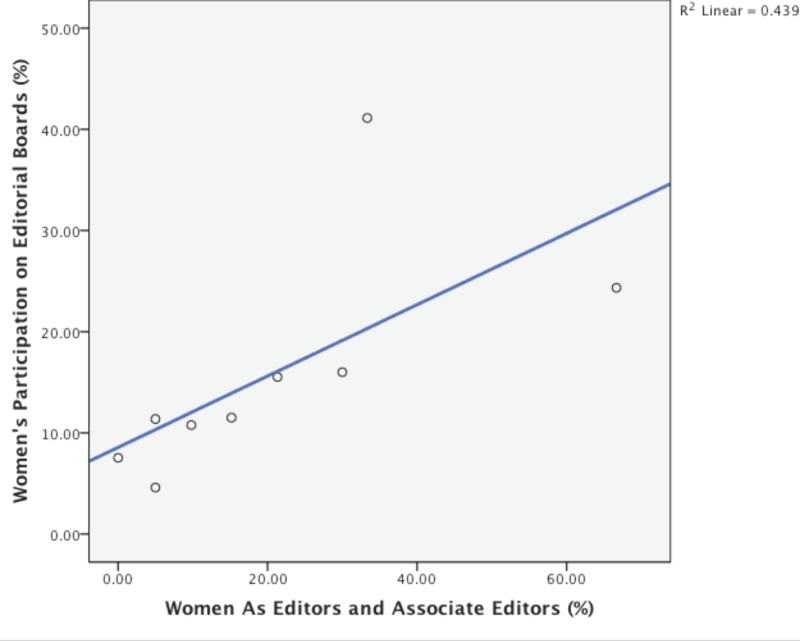
The association of women on editorial leadership and advisory board membership. The scatterplot and line fit shows a linear trend (r^2^ = 0.44) and a significant correlation between women on journal editorial leadership and women as editorial and advisory board members. (p = 0.03).

When editors-in-chief and associate editors-in-chief were combined and defined as the journal editorial leadership, we found that it positively and significantly correlated with the number of women in editorial leadership and the number of women represented as advisory board members (p = 0.03, r^2^ = 0.44) (Fig. 4).

## Discussion

Our results highlight the under-representation of women on editorial and advisory boards of major dental journals, which was, in most cases, proportional to their representation in academic disciplines. More specifically, journals in the field of periodontology, followed by oral and maxillofacial surgery, showed the lowest participation of women on editorial boards (4.6% and 7.5%, respectively), whereas journals in public health showed the highest presence of women on their boards (41.1%).

An examination of journal editorial leadership found that only two of the 69 journals had women serving as editors-in-chief. Furthermore, we found that only 16% of the boards had women serving as associate editors-in-chief. Surprisingly, the journals with a female editor-in-chief did not result in more women as advisory board members.

Similar studies performed in the medical field showed 11.5–17.5% representation of women on medical journal editorial and advisory boards [9,11,13,14]. In 7.0–15.9% of the major medical journals women served as editors-in-chief [9,11] as opposed to mere 2.5% in the dental journals examined by the present study.

In certain specialties (endodontics, oral and maxillofacial surgery, public health, pediatric dentistry), women’s participation on editorial boards reached parity with the corresponding academic presence in the dental specialty, which may reflect the proactive efforts to promote women. Interestingly, in periodontology, although women represented 22% of academic faculty, they only held 4.6% of the editorial board positions. The rest of specialties (orthodontics and prosthodontics as well as general dentistry) showed some disparities in the range of 6–11% between the percentage of women in an academic position and percentage of women in editorial board positions (Table 1). The cause of the under-representation of women on editorial boards may be due to unconscious psychological factors, which results in men being promoted over women [2]. Due to the absence of institutional support, women tend to be more focused on teaching or clinical activities as opposed to research, resulting in women receiving less scholarly recognition [21]. As a result, fewer women are promoted to senior academic positions and fewer receive the honors and awards, which come from scholarly productivity [2,22,23]. Moreover, institutional leadership frequently is influenced by traditional gender roles and expectations, resulting in barriers for the advancement of women [22]. Women currently represent 45% of the dental students, 21% of the professionally active dentists [24] but they hold only 28% of the dental academic positions and 19% of the dental school dean positions [25] which confirms that women still face barriers in the promotion process. Collectively, the above factors may certainly limit the editorial and advisory board invitations to women academicians.

The study inherited some limitations due to its cross-sectional design, which prevented any longitudinal trend assessment. Furthermore, when comparing the effect of gender in the editor-in-chief position to the overall composition of the editorial board, the sample was very small such that only limited analyses were possible. Evidence has shown that throughout the last 20 years, the participation of women on medical journal editorial boards has been improving [9,26]. However, the present cross-sectional study identified a significant under-representation of women on editorial/advisory boards in dentistry. A larger longitudinal editorial board analysis is recommended to investigate the trend of women’s participation in editorial board of dental journals during recent decades.

Several steps could be undertaken to change the current state of under participation of women. In 2012, the European Association of Science Editors established a “Gender Policy Committee” with the goal to “encourage gender balance among reviewers, editorial boards and editorial offices” [27]. The National Academy of Science has published recommendations and criteria for journals in order to achieve gender diversity [1]. More specifically, they have recommended the development and enforcement of guidelines, which will “ensure significant representation of women on meeting speaker lists, on editorial boards, and in other significant leadership positions”. For women academicians, these measures will create more opportunities for career development and success. Additionally, the academic institutions will need to recognize the disparities, mentor women, as well as nominate them to intramural and extramural leadership positions. Together with the above, women scientists may need to “lean in” and proactively inquire about editorial and advisory board positions. These collective actions may alter the current atmosphere in the journal editorial board leading to increased diversity.
